# High-level resistance to aminoglycosides and ampicillin among clinical isolates of *Enterococcus* species in an Iranian referral hospital

**DOI:** 10.18502/ijm.v12i4.3935

**Published:** 2020-08

**Authors:** Seyed Hossein Mousavi, Hadi Peeri-Doghaheh, Behnam Mohammadi-Ghalehbin, Roghayeh Teimourpour, Dadras Maleki, Farzad Khademi, Mohsen Arzanlou

**Affiliations:** 1 Department of Microbiology, School of Medicine, Ardabil University of Medical Sciences, Ardabil, Iran; 2 Microbiology Laboratory, Imam Hospital, Ardabil University of Medical Sciences, Ardabil, Iran

**Keywords:** *Enterococcus faecalis*, *Enterococcus faecium*, High-level resistance, Gentamicin, Streptomycin, Ampicillin

## Abstract

**Background and Objectives:**

Nowadays, high-level aminoglycosides and ampicillin resistant *Enterococcus* species are among the most common causes of nosocomial infections. The present study was conducted to determine the prevalence of high-level resistance to aminoglycosides and ampicillin among clinical isolates of *Enterococcus* species in Ardabil, Iran.

**Materials and Methods:**

In this cross–sectional study, a total of 111 *Enterococcus* species were collected from different clinical specimens between 2013 and 2015. *Enterococcus* species were identified using standard phenotypic and genotypic methods. BHI agar screen and agar dilution methods were used for detection of high-level gentamicin and streptomycin resistance (HLGR and HLSR) and minimal inhibitory concentration (MIC) of ampicillin, respectively.

**Results:**

Of 111 clinical isolates, 59 (53.2%) and 25 (22.5%) isolates were *E. faecalis* and *E. faecium*, respectively, based on the PCR results. Totally, 60.3% and 56.7% of isolates were HLGR and HLSR, respectively, as well as 51.35% were HLGR plus HLSR. Among HLGR isolates, 36 (61.01%), 18 (72%) and 13 (48.14%) were *E. faecium, E. faecalis* and non-*faecalis* non-*faecium* species, respectively. Among HLSR isolates, 33 (55.93%), 16 (64%) and 14 (51.85%) were *E. faecalis, E. faecium* and non-*faecalis* non-*faecium* species, respectively. All HLGR isolates contained *aac(6′)Ie-aph(2″)Ia* gene. Overall, the prevalence of high-level ampicillin resistance among *Enterococcus* species was 17.1%. For *E. faecalis, E. faecium* and non-*faecalis* non-*faecium* species, ampicillin resistance rates were as follows: 11 (40.74%), 7 (28%) and 1 (1.69%), respectively. For aminoglycoside antibiotics, the resistance rate was significantly higher in *E. faecium* isolates and for ampicillin it was higher in *E. faecalis* isolates.

**Conclusion:**

The frequency of high-level aminoglycoside resistant enterococcal isolates in our hospital was high and significant ampicillin resistance was noticed. This would require routine testing of enterococcal isolates for HLAR and ampicillin susceptibility.

## INTRODUCTION

*Enterococcus* species have been ranked as the second to third most common organisms responsible for nosocomial infections, especially in critically ill patients or individuals who received multiple antibiotics (1). In clinical practice, most of non-invasive and uncomplicated enterococcal infections are usually cured using a single antibiotic regimen. While, to achieve an efficient bactericidal activity, combination therapy is needed for treating deep-seated enterococcal infections (such as endocarditis) (2). The most common therapy regimen is a combination of a cell wall active agent (e.g., ampicillin and vancomycin) and an aminoglycoside, gentamicin or streptomycin (2). These agents act synergistically to enhance killing of the bacteria, since the beta-lactam agents damage the cell wall and increase the entry of the aminoglycosides into the cell (3). Generally, penicillins (e.g., ampicillin) have the highest activity, carbapenems slightly lower and cephalosporins and aztreonam have the lowest activity (4). *Enterococcus* spp. show low-level intrinsic resistance against beta-lactam and aminoglycoside antibiotics (3). The major categories of acquired antibiotic resistance in enterococci include high-level penicillin and ampicillin resistance, high-level aminoglycoside resistance (HLAR) and vancomycin resistance (3, 4). The acquisition of these types of resistance eliminates the synergistic effect of combination therapy (2).

The aim of this study was to determine the frequency of high-level resistance to aminoglycosides and ampicillin among enterococcal isolates collected from different clinical specimens in an Iranian hospital.

## MATERIALS AND METHODS

### Media and chemicals.

DNP™ Genomic DNA Extraction Kit was purchased from Cinagen Company (Cinagen, Iran). AccuPower™ PCR PreMix Kit and oligonucleotide primers were obtained from Bioneer Company (Daejon, South Korea). Gentamicin, streptomycin and ampicillin powders were purchased from Bio Basic Company (Bio Basic Inc. Canada). Brain Hart Infusion Agar (BHI) was obtained from Difco Laboratories (Detroit, MI, USA) and Trypticase Soy Broth (TSB), Blood Agar, Bile Esculin Agar (BEA) and Mueller Hinton Agar (MHA) were purchased from Himedia laboratories (Mumbai, India).

### Isolation and identification of bacteria.

From November 2013 to September 2015, a total of 111 enterococci strains were isolated from different clinical specimens collected from patients admitted to a referral teaching hospital affiliated to Ardabil University of Medical Sciences. Isolates were examined by conventional phenotypic methods at the genus level. *E. faecium* and *E. faecalis* were identified using PCR analysis of the *ddl* gene as described previously (1, 5). *E. faecalis* ATCC 29212 and *E. faecium* ATCC 51559 were used as controls. Identified isolates were stored at −80ºC in TSB containing 15% glycerol.

### Antimicrobial susceptibility testing.

High-level aminoglycosides resistance (HLAR), gentamicin (HLGR) and streptomycin (HLSR), were determined using BHI agar screen method (1). Testing for minimum inhibitory concentration (MIC) of ampicillin was performed by standard agar dilution method (concentration rang: 0.12–1024 μg/ml) (1). Resistance to ampicillin was defined as MIC ≥ 16 (μg/ml).

Antimicrobial susceptibility testing was performed and interpreted according to the guidelines of the Clinical and Laboratory Standards Institute (CLSI) (6). *Enterococcus faecalis* ATCC 29212 was used as control strain.

### PCR amplification of HLGR resistance gene.

The presence of *aac(6′)Ie-aph(2″)Ia* gene in the genome of HLGR isolates was detected using PCR method as reported earlier (10, 12).

### Statistical analysis.

Chi-square test was used to compare the prevalence of HLAR strains between specimen type and hospital wards.

## RESULTS

In the present study, a total of 111 *Enterococcus* species were isolated from different clinical specimens from patients who referred to Imam Khomeini Hospital in Ardabil province. Totally, 61 (54.9%) and 50 (45.1%) isolates were collected from female and male patients, respectively. Enterococci isolates were collected from urine (n=87), blood (n=21), wound (n=2) and sputum (n=1) specimens.

### PCR analysis of *ddl* gene.

Identification of *E. faecalis* and *E. faecium* species was performed by amplification of *ddl* gene using PCR method. According to the PCR results, 59 (53.2%) and 25 (22.5%) isolates were *E. faecalis* and *E. faecium*, respectively, as well as 27 (24.3%) isolates belonged to non-*faecalis* non-*faecium Enterococcus* species.

### Detection of HLGR and HLSR strains.

**Fig. 1.**
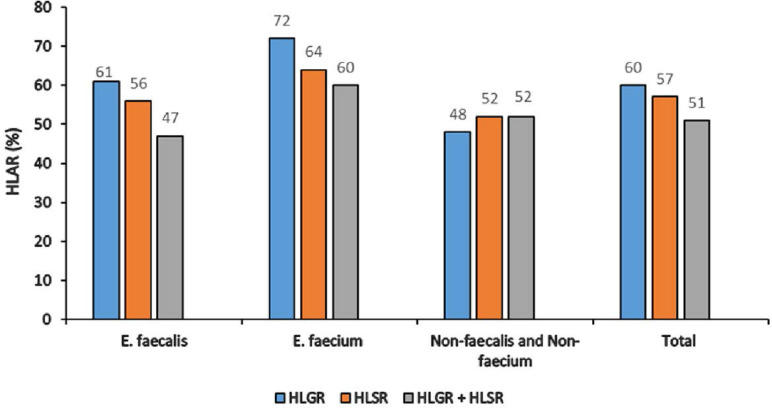
Prevalence of HLAR among *Enterococcus* species isolated from clinical specimens HLAR; High level aminoglycoside resistance, HLGR; High level gentamicin resistance, HLSR; High level streptomycin resistance

Totally, 60.3% and 56.7% of isolates were HLGR and HLSR, respectively, as well as 51.35% were HLGR plus HLSR. Among HLGR isolates, 36 (61.01%), 18 (72%) and 13 (48.14%) were *E. faecium, E. faecalis* and non-*faecalis* non-*faecium* species, respectively. Among HLSR isolates, 33 (55.93%), 16 (64%) and 14 (51.85%) were *E. faecalis, E. faecium* and non-*faecalis* non-*faecium* species, respectively. Statistically, there was no significant difference between the prevalence of HLGR and HLSR phenotypes in *Enterococcus* spp. (P > 0.05) (Fig. 1). All HLGR isolates were contained the *aac(6′)Ie-aph(2″)Ia* gene.

The prevalence of HLGR and HLSR phenotypes was significantly different according to the hospital wards in both *E. faecium* and *E. faecalis* species (P < 0.001). The majority of them were isolated from patients who admitted to internal medicine ward, infectious diseases ward and outpatient clinic, respectively (Table 1). However, statistically there was no significant difference in distribution of *Enterococcus* spp. between wards, except for HLGR *E. faecium* isolates in infectious disease ward and HLGR and HLSR non-*faecalis* non-*faecium* species in internal medicine ward (P < 0.05).

**Table 1.** Prevalence of HLAR among *Enterococcus* species isolated from clinical specimens in different hospital ward

	**HLAR**					
	**HLGR**			**HLSR**		
	
**Hospital ward**	***E. faecalis* (n = 36) n (%)**	***E. faecium* (n = 18) n (%)**	**non-*faecalis* non-*faecium* (n = 13) n (%)**	***E. faecalis* (n = 33) n (%)**	***E. faecium* (n = 16) n (%)**	**non-*faecalis* non-*faecium* (n = 14) n (%)**
Internal medicine	13 (36.11)	9 (50)	9(69.23 )^*^	12 (36.37)	7 (43.75)	9 (64.30)^*^
ICU	3 (8.33)	1 (5.6)	-	3 (9.10)	2 (12.5)	-
Infectious disease	6 (16.70)	4 (22.20)^*^	1(7.7)	6 (18.2)	3 (18.75)	2 (14.30)
Outpatient clinic	6 (16.70)	2 (11.10)	2(15.40)	6 (18.2)	2 (12.5)	2 (14.30)
Emergency	3 (8.33)	1 (5.6)	-	3 (9.10)	1 (6.25)	-
Cardiology	2 (5.55)	-	-	1 (3.03)	-	-
Cardiothorax	1 (2.80)	-	1(7.7)	1 (3.03)	-	1 (7.14)
Coronary care unit (CCU)	1 (2.77)	1 (5.6)	-	1 (3.03)	1 (6.25)	-

* Statistically significant (P < 0.05)

HLAR; High level aminoglycoside resistance, HLGR; High level gentamicin resistance, HLSR; High level streptomycin resistance

The majority of HLAR *Enterococcus* species were isolated from urine specimens (P < 0.001). However, there was no significant difference between distribution of HLAR *Enterococcus* species and specimen type (P > 0.05) (Table 2).

**Table 2.** Prevalence of HLAR among *Enterococcus* species isolated from clinical specimens based on specimen type

	**HLAR**					
	**HLGR**			**HLSR**		

**Specimen type**	***E. faecalis* (n = 36) n (%)**	***E. faecium* (n = 18) n (%)**	**non-*faecalis* non-*faecium* (n = 13) n (%)**	***E. faecalis* (n = 33) n (%)**	***E. faecium* (n = 16) n (%)**	**non-*faecalis* non-*faecium* (n = 14) n (%)**
Urine	31 (86.10)	15 (83.33)	11 (84.61)	27 (81.80)	12 (75)	11 (78.60)
Blood	4 (11.11)	3 (16.66)	2 (15.40)	5 (15.15)	4 (25)	3 (21.43)
Wound	1 (2.80)	-	-	1 (3.00)	-	-

HLAR; High level aminoglycoside resistance, HLGR; High level gentamicin resistance, HLSR; High level streptomycin resistance

### Determining MIC for ampicillin.

In this study, MIC of ampicillin was determined using agar dilution method. Totally, 19 (17.1%) out of 111 enterococci isolates showed high-level resistance to ampicillin (MIC ≥ 16 μg/mL). Ampicillin resistance in *E. faecalis* isolates was found in 11 (18.6%) out of 59 isolates. The MIC values for ampicillin resistant *E. faecalis* isolates were ranged from 32–512 μg/mL [32; 1 (9.10%), 64; 1 (9.10%), 256; 4 (36.36%) and 512; 5 (45.45%)]. Twenty-eight percent (7/25) of *E. faecium* isolates with MIC values from 32 to 1024 μg/mL [32; 2 (28.57%), 64; 1 (14.28%), 256; 1 (14.28%), 512; 4 (45.45%) and 1024; 1 (14.28%)] were found to be ampicillin resistant. In non-*faecalis* and non-*faecium* enterococci 3.6% of isolates were ampicillin resistant (MIC; 32 μg/mL).

## DISCUSSION

Nowadays, acquired high-level ampicillin resistance in *Enterococcus* species is an increasing global concern. Epidemiology of enterococci resistant to ampicillin varies among hospitals and countries. In the USA and Europe, the majority of nosocomial invasive enterococci isolates are resistant to ampicillin (7). Ampicillin resistance is most commonly seen in *E. faecium* isolates (7). Accordingly, in this study, resistance to ampicillin in *E. faecium* (28%) was higher than *E. faecalis* (19%) isolates. Previously it has been shown that 90%, 84%, 74% and 69% of *E. faecium* recovered from healthcare-associated infections in the USA, Argentina, Denmark and Iran were ampicillin resistant, respectively (4, 7–9). In Ardabil, the prevalence of ampicillin-resistant *E. faecium* isolates was significantly lower than the other parts of the world as well as Iran (15). Interestingly, the prevalence of ampicillin-resistant *E. faecalis* isolates was high (19%) in this study which is similar to Asadollahi et al. report from Iran (17.1%) (15). Given the negligible incidence of ampicillin-resistant *E. faecalis* isolates in other parts of the world, our findings represent a significant increase in ampicillin resistance rate in *E. faecalis* isolates in Iran. In a report from Argentina in 2015, ampicillin resistance rate was only 1.8% in *E. faecalis* isolates (4). While, high-level resistance to penicillin and aminopenicillins have not yet been described in non-*faecalis* non-*faecium Enterococcus* species (4), in this study one non-*faecalis* non-*faecium* isolate was found to be resistant against ampicillin.

Recently several antibiotics were introduced to be used in combination with ampicillin in the treatment of enterococcal invasive infections (2). However, aminoglycoside antibiotics are still the main component of combination therapy of enterococcal invasive infections (2). With the emergence of high-level resistance, any synergistic effect between aminoglycosides and cell wall active agents is lost. In recent years, acquired high-level resistance to aminoglyco-sides increased significantly by the wide spread of genes encoding aminoglycoside modifying enzymes (AMEs). In a survey conducted in Australia in 2013, 32.4% of *E. faecalis* and 61.8% of *E. faecium* isolates showed HLGR phenotype (10). Because of limited use of streptomycin, very few studies have recently studied prevalence of HLSR phenotype among *Enterococcus* species. In a twelve-year surveillance in Japan, HLSR phenotype was detected in 18% of *E. faecalis* and 39% of *E. faecium* isolates in 2016 (11). Unfortunately, there is no a nationwide surveillance data for HLGR and HLSR phenotypes in Iran. However, sporadic studies were reported a HLGR phenotype range of 33 to 77.3% in *E. faecium* and 25.84% to 89% in *E. faecalis* isolates. Similarly, HLSR phenotype was reported as 27.27% to 90.10% and 40.44% to 73.10% in *E. faecium* and *E. faecalis* isolates, respectively (12–16). In the current study, the prevalence of HLGR was observed among 61.01% of *E. faecalis*, 72% of *E. faecium*, and 48.14% of non-*faecalis* non-*faecium* species. Additionally, 33 (55.93%) of *E. faecalis*, 16 (64%) of *E. faecium* and 14 (51.85%) of non-*faecalis* non-*faecium* species had HLSR phenotype. Similar to other reports around the world (11), the proportion of HLGR and HLSR were higher in *E. faecium* compared to *E. faecalis* isolates. In this study, 45.47% and 60% of *E. faecalis* and *E. faecium* isolates exhibited dually high-level gentamicin and streptomycin resistance, respectively, which is similar to findings reported in other studies (15). As high-level resistance to gentamicin and streptomycin is caused by different mechanisms (3), these antibiotics can be used surrogate to each other in enterococcal infections treatment. Therefore, the emergence of isolates with simultaneous resistance phenotype, HLGR and HLSR, will limit the therapeutic options of enterococcal infections.

High-level aminoglycosides resistance is primarily due to the presence of the AMEs. In *Enterococcus* species, there are several AMEs responsible for HLGR and HLSR (1). The *aac(6′)Ie-aph(2′)Ia* gene is the most prevalent gene encoding a AME which causes high-level resistance to aminoglycosides except for streptomycin (1). In the present study, we examined only the presence of the *aac(6′)Ie-aph(2′)* Ia gene for strains with HLGR. Our findings showed that all HLGR isolates were positive for *aac(6′)Ieaph(2′)Ia* gene. Similar results were reported from Iran and other countries (11, 14). Genetic basis for HLSR was not further studied in this study.

## CONCLUSION

The frequency of high-level aminoglycoside resistant enterococcal isolates in our hospital was high and significant ampicillin resistance was noticed. Therefore, to achieve an optimal therapeutic outcome, continuous monitoring is needed through routine susceptibility testing of enterococcal isolates against aminoglycosides and ampicillin.

## References

[1] Jannati E Amirmozaffari N Saadatmand S Arzanlou M. Faecal carriage of high-level aminoglycoside-resistant and ampicillin-resistant *Enterococcus* species in healthy Iranian children. J Glob Antimicrob Resist 2020; 20:135–144. 31295581 10.1016/j.jgar.2019.06.022

[2] Fraser Susan L Enterococcal infections treatment and management. 2018. Available from https://emedicine.medscape.com/article/216993-treatment

[3] Hollenbeck BL Rice LB. Intrinsic and acquired resistance mechanisms in *Enterococcus*. Virulence 2012; 3:421–433. 23076243 10.4161/viru.21282 PMC3485979

[4] Gagetti P Bonofiglio L García Gabarrot G Kaufman S Mollerach M Vigliarolo L Resistance to β-lactams in enterococci. Rev Argent Microbiol 2019; 51: 179–183. 30243525 10.1016/j.ram.2018.01.007

[5] Dutka-Malen S Evers S Courvalin P. Detection of glycopeptide resistance genotypes and identification to the species level of clinically relevant enterococci by PCR. J Clin Microbiol 1995;33:24–27. 7699051 10.1128/jcm.33.1.24-27.1995 PMC227872

[6] Clinical and Laboratory Standards Institute. Performance standards for antimicrobial susceptibility testing. 26th ed CLSI supplement M100s. CLSI; 2016.

[7] Hidron AI Edwards JR Patel J Horan TC Sievert DM Pollock DA NHSN annual update: antimicrobial-resistant pathogens associated with healthcare-associated infections: annual summary of data reported to the national healthcare safety network at the centers for disease control and prevention, 2006–2007. Infect Control Hosp Epidemiol 2008; 29: 996–1011. 18947320 10.1086/591861

[8] Lester CH Sandvang D Olsen SS Schønheyder HC Jarløv JO Bangsborg J Emergence of ampicillin-resistant *Enterococcus faecium* in Danish hospitals. J Antimicrob Chemother 2008; 62:1203–1206. 18765412 10.1093/jac/dkn360

[9] Asadollahi P Razavi S Asadollahi K Pourshafie MR Talebi M. Rise of antibiotic resistance in clinical enterococcal isolates during 2001–2016 in Iran: a review. New Microbes New Infect 2018; 26: 92–99. 30319780 10.1016/j.nmni.2018.08.018 PMC6180340

[10] Coombs GW Pearson JC Daley DA Le TT Robinson JO Gottlieb T Australian enterococcal sepsis outcome programme annual report, 2013. Commun Dis Intell Q Rep 2014;38:E320–326. 25631594 10.33321/cdi.2014.38.52

[11] Osuka H Nakajima J Oishi T Funayama Y Ebihara T Ishikawa H High-level aminoglycoside resistance in *Enterococcus faecalis* and *Enterococcus faecium* causing invasive infection: Twelve-year surveillance in the Minami Ibaraki Area. J Infect Chemother 2016; 22:61–63. 26492859 10.1016/j.jiac.2015.09.003

[12] Khodabandeh M Mohammadi M Abdolsalehi MR Hasannejad-Bibalan M Gholami M Alvandimanesh A High-level aminoglycoside resistance in *Enterococcus faecalis* and *Enterococcus faecium*; as a serious threat in hospitals. Infect Disord Drug Targets 2020;20:223–228. 30499420 10.2174/1871526519666181130095954

[13] Khani M Fatollahzade M Pajavand H Bakhtiari S Abiri R. Increasing prevalence of aminoglycoside-resistant *Enterococcus faecalis* isolates due to the *aac(6′)-aph(2″)* gene: A therapeutic problem in Kermanshah, Iran. Jundishapur J Microbiol 2016; 9(3): e28923. 27217920 10.5812/jjm.28923 PMC4870677

[14] Dadfarma N Fooladi AAI Oskoui M Mahmoodzadeh Hosseini H. High level of gentamicin resistance (HLGR) among enterococcus strains isolated from clinical specimens. J Infect Public Health 2013;6:202–208. 23668465 10.1016/j.jiph.2013.01.001

[15] Behnood A Farajnia S Moaddab SR Ahdi-Khosroshahi S Katayounzadeh A. Prevalence of *aac(6′)-Ieaph(2″)-Ia* resistance gene and its linkage to Tn5281 in *Enterococcus faecalis* and *Enterococcus faecium* isolates from Tabriz hospitals. Iran J Microbiol 2013;5:203–208. 24475324 PMC3895555

[16] Emaneini M Aligholi M Aminshahi M. Characterization of glycopeptides, aminoglycosides and macrolide resistance among *Enterococcus faecalis* and *Enterococcus faecium* isolates from hospitals in Tehran. Pol J Microbiol 2008; 57:173–178. 18646406

